# Promotion effect of extracts from plastrum testudinis on alendronate against glucocorticoid-induced osteoporosis in rat spine

**DOI:** 10.1038/s41598-017-10614-5

**Published:** 2017-09-06

**Authors:** Hui Ren, Gengyang Shen, Jingjing Tang, Ting Qiu, Zhida Zhang, Wenhua Zhao, Xiang Yu, Jinjing Huang, De Liang, Zhensong Yao, Zhidong Yang, Xiaobing Jiang

**Affiliations:** 1 Department of Spinal Surgery, The First Affiliated Hospital of Guangzhou University of Chinese Medicine Guangzhou, Guangzhou, 510405 China; 2 Guangzhou University of Chinese Medicine Guangzhou , Guangzhou, 510405 China; 3 Laboratory Affiliated to National Key Discipline of Orthopaedic and Traumatology of Chinese Medicine, Guangzhou University of Chinese Medicine, Guangzhou, 510405 China

## Abstract

Alendronate (ALN) is a key therapeutic used to treat glucocorticoid-induced osteoporosis (GIOP), but may induce severe side effects. We showed earlier that plastrum testudinis extracts (PTE) prevented and treated GIOP *in vivo*. However, clinically, PTE is seldom used alone. Herein, we reveal the synergistic effect of ALN and PTE can treat GIOP of the rat spine and define the mechanism. Sprague-Dawley rats were randomly assigned to four groups: a vehicle group, a GIOP group, an ALN group, and an ALN+PTE group. Each group was further divided into two experimental phases, including dexamethasone (DXM) intervention and withdrawal. Bone mass, microarchitecture, biomechanics, bone-turnover markers, and histomorphology were evaluated. The mRNA and protein expression levels of CTSK and Runx2 were detemined. We found that ALN+PTE improved bone quantity and quality, bone strength, bone turnover; and mitigated histological damage during glucocorticoid intervention and withdrawal. The therapeutic effect was better than that afforded by ALN alone. ALN+PTE reduced CTSK protein expression, promoted Runx2 mRNA and protein expression to varying extents, and more strongly inhibited bone resorption than did ALN alone. Overall, the synergistic effect mediated by ALN+PTE reversed GIOP during DXM intervention and withdrawal via affecting CTSK and Runx2 expression at mRNA and protein levels.

## Introduction

Substantial progress has been made in our understanding of the pathogenesis of glucocorticoid-induced osteoporosis (GIOP), the most common form of secondary osteoporosis^1–3^. However, effective treatment remains challenging^4^. The current international guidelines recommend the use of bisphosphonates, such as alendronate (ALN) which inhibit bone resorption, to prevent and treat patients with, or at risk of, GIOP^5–8^. ALN can induce severe side-effects such as rash, weight loss, sciatica, and asthma, etc^9^. Therefore, innovative anti-GIOP strategies are required.

Natural products serve as the sources of, and inspiration for, many current drugs. Plastrum testudinis (PT) is an important traditional Chinese medicine that is often used to treat bone diseases in China. In previous studies^10–12^, plastrum testudinis extracts (PTE) were shown to promote osteoblastic function *in vitro*, and to prevent and treat GIOP *in vivo*. However, clinically, PTE alone are seldom used to treat bone diseases. Thus, in the present study, we explore whether PTE acted synergistically with ALN to counter GIOP in terms of bone quality and quantify, expression of bone turnover markers, and bone histology. We describe the mechanism of the synergistic effect found.

Previously^13^, we confirmed that glucocorticoid (GC) withdrawal for 3 months did not reduce bone impairment in rats with GIOP; we considered that, clinically, continued anti-osteoporosis treatment was required after GC withdrawal. However, basic studies are obviously required before a new therapeutic strategy can be clinically applied. Thus, herein, we used a 3-months course of ALN+PTE to treat rats with GIOP after GC withdrawal; we assessed changes in bone quality and quantify, the expression of bone turnover markers, and bone histology. We also defined the mechanism of action in play.

To explore the synergistic effect of PTE and ALN on GIOP in detail, rats received oral ALN+PTE both at the time of GC injection and withdrawal. Bone mineral density (BMD), bone mineral content (BMC), bone area (AREA), bone microarchitecture, bone biomechanics, bone metabolism, and bone histomorphology (as revealed by haematoxylin and eosin [HE], and tartrate-resistant acid phosphastase [TRAP], staining) were evaluated. The mRNA and protein expression levels of Cathepsin K (CTSK) and Runx2 were determined to identify the potential therapeutic targets of ALN+PTE used to treat GIOP.

## Results

### ALN+PTE increases bone quantity during both the DXM intervention and withdrawal periods

**Figure 1:**
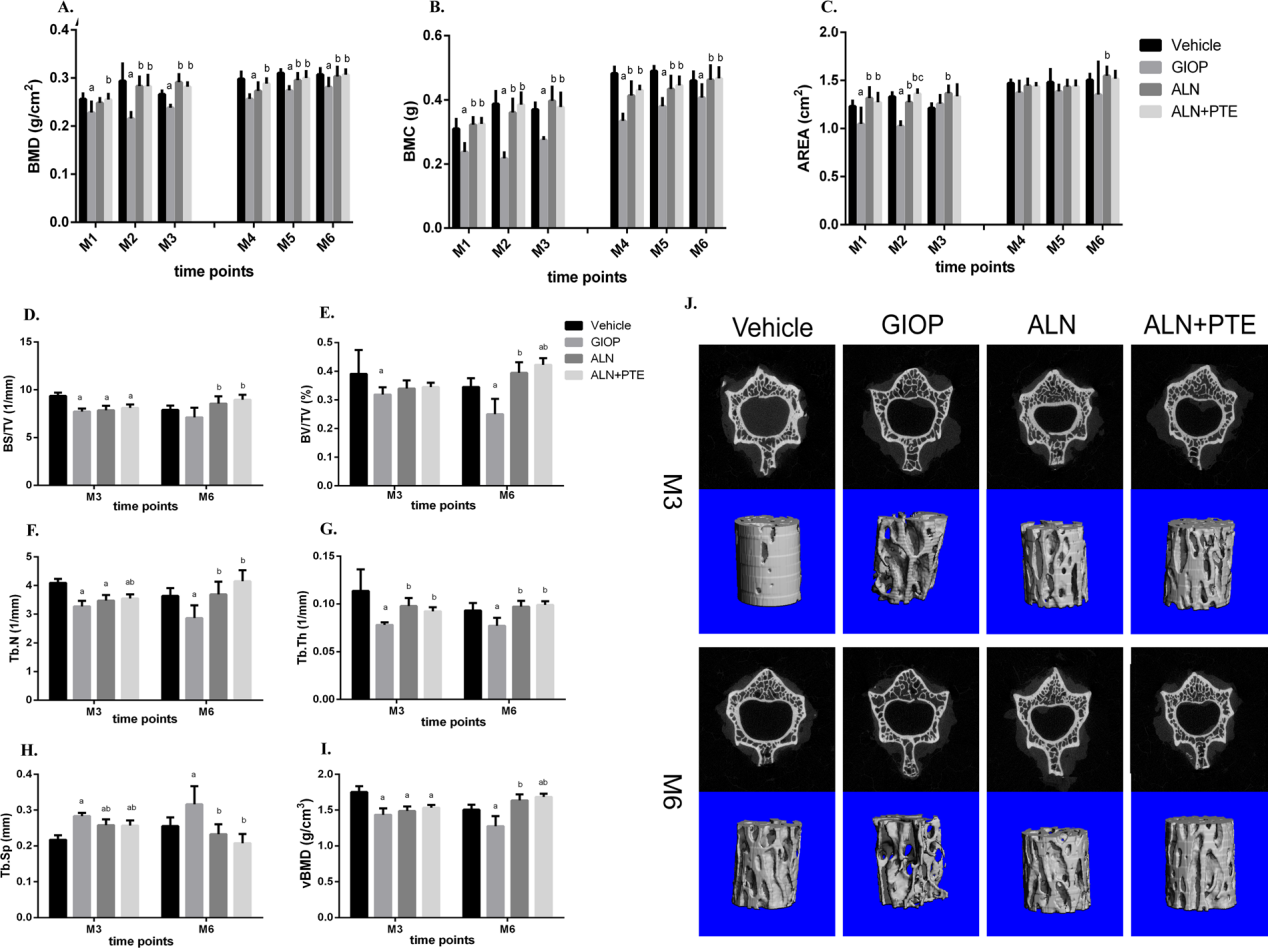
(**A**–**C**) Changes in bone mineral density (BMD), bone mineral content (BMC) and bone area (AREA) of L1-3. (**D–I**) Effects of DXM intervention, DXM withdrawal, ALN treatment and ALN+PTE treatment on parameters determined by micro-CT. (**J**) Changes in L2 microarchitecture of rats. The upside shows two-dimensional images, and the underside shows three-dimensional images. Values are the means ± SD. ^a^ *P* < 0.05 vs the vehicle group; ^b^ *P* < 0.05 vs the GIOP group; ^c^ *P* < 0.05 vs the ALN group.

During the DXM intervention period (months M1-M3), the GIOP group (compared to the vehicle group) exhibited significantly lower BMDs and BMCs at each time-point and significantly reduced AREAs at both M1 and M3. Compared with the GIOP group, the ALN+PTE group exhibited significantly higher BMDs and BMCs at each time-point and greater AREAs at both M1 and M2. We found no significant difference in any of BMD, BMC, or AREA between ALN and ALN+PTE groups (Fig. 1A–C).

During the DXM withdrawal period (M4-M6), the GIOP group (compared to the vehicle group) exhibited significantly lower BMDs and BMCs at each time-point. Compared with the GIOP group, the ALN+PTE group exhibited significantly higher BMDs and BMCs at each time-point. Compared with the ALN group, the ALN+PTE group exhibited higher BMDs and BMCs at each time-point, although the difference were not statistically significant (Fig. 1A–C).

### ALN+PTE improves bone quality during both the DXM intervention and withdrawal periods

Figure 1(D–I) shows L2 trabecular bone microarchitecture data determined by micro computed tomography (CT). After DXM intervention (at M3), the GIOP group (compared to the vehicle group) exhibited a significantly lower relative bone surface (BS/TV), relative bone volume (BV/TV), trabecular number (Tb.N), trabecular thickness (Tb.Th), and volume bone mineral density (vBMD); and a higher trabecular separation (Tb.Sp). Compared with the GIOP group, the ALN+PTE group exhibited a significantly higher Tb.N and Tb.Th, and a lower Tb.Sp. Compared with the ALN group, the ALN+PTE group exhibited a higher BS/TV, BV/TV, Tb.N, and vBMD, although the differences were not statistically significant.

After DXM withdrawal (at M6), the GIOP group (compared to the vehicle group) exhibited a significantly lower BV/TV, Tb.N, Tb.Th, and vBMD; and a higher Tb.Sp. Compared with the GIOP group, the ALN+PTE group exhibited a significantly higher Tb.N and Tb.Th, and a lower Tb.Sp. Compared with the ALN group, the ALN+PTE group exhibited a higher BS/TV, BV/TV, Tb.N, and vBMD; and a lower Tb.Sp, although the differences were not statistically significant (Fig. 1D–I).

At the M3 and M6 time-points, both two- and three-dimensional images revealed small amounts of thin, impaired trabecular bone in the GIOP group; the extent of such bone was significantly less in the ALN+PTE group. Also, the ALN+PTE group had more trabecular and exhibited a denser distribution of trabecular bone (Fig. 1J).

### ALN+PTE increases bone strength during both the DXM intervention and withdrawal periods

**Figure 2:**
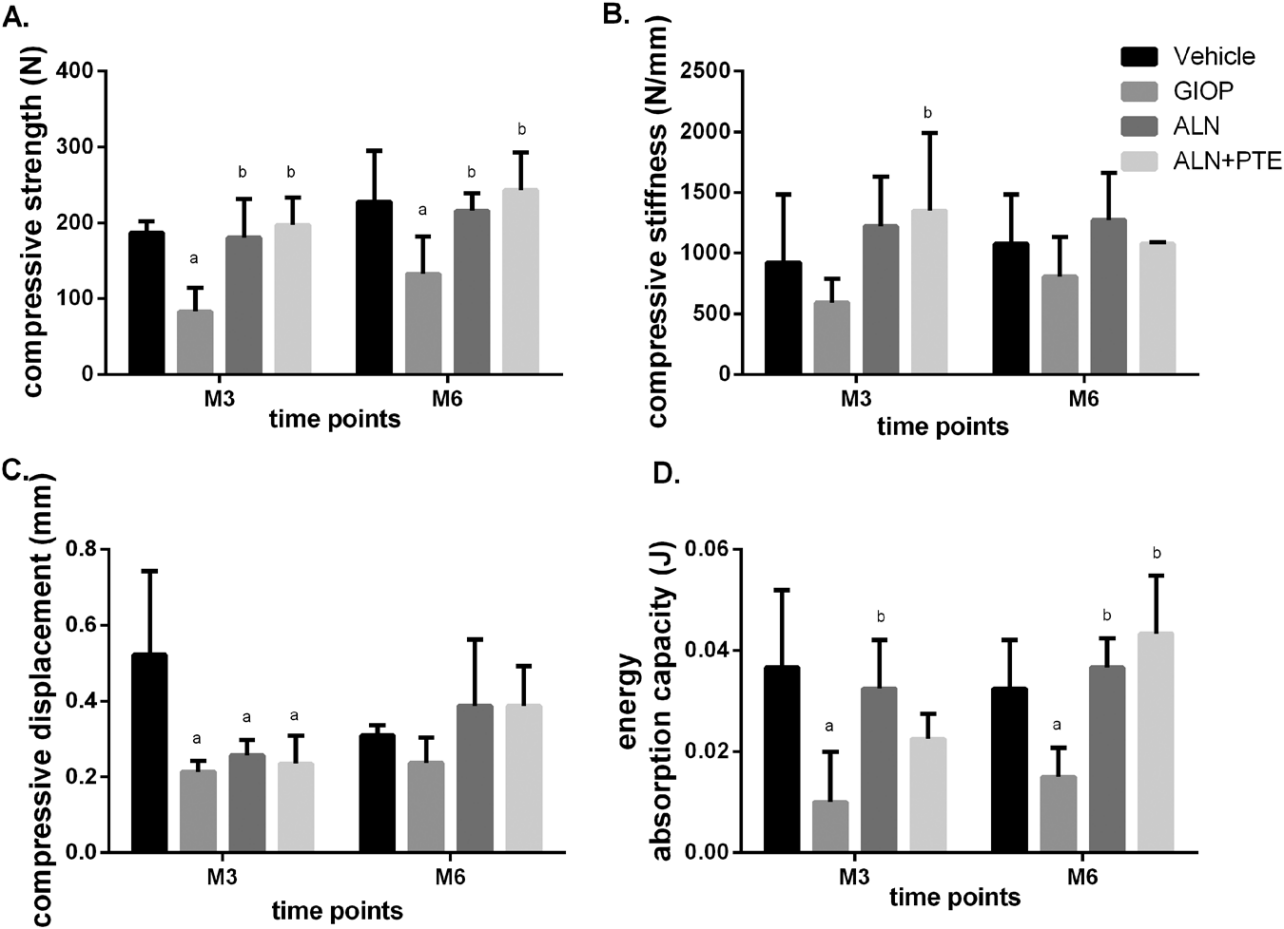
Results of biomechanical analysis of L2. Values are the means ± SD. ^a^ *P* < 0.05 vs the vehicle group; ^b^ *P* < 0.05 vs the GIOP group; ^c^ *P* < 0.05 vs the ALN group.

Figure 2 shows the changes in bone strength, bone stiffness, compressive displacement, and energy absorption capacity. After DXM intervention (at M3), the GIOP group (compared to the vehicle group) exhibited significantly reductions in compressive strength, energy absorption capacity, and compressive displacement. Compared with the GIOP group, the ALN+PTE group exhibited significantly higher compressive strength and compressive stiffness. Compared with the ALN group, the ALN+PTE group exhibited higher compressive strength and compressive stiffness, although the differences were not significant.

After DXM withdrawal (at M6), the GIOP group (compared to the vehicle group) exhibited significantly reductions in compressive strength and energy absorption capacity. Compared with the GIOP group, the ALN+PTE group exhibited significantly higher compressive strength and energy absorption capacity. Compared with the ALN group, the ALN+PTE group exhibited higher compressive strength and energy absorption capacity, although the differences were not significant (Fig. 2).

### ALN+PTE improves bone turnover during both the DXM intervention and withdrawal periods

**Figure 3:**
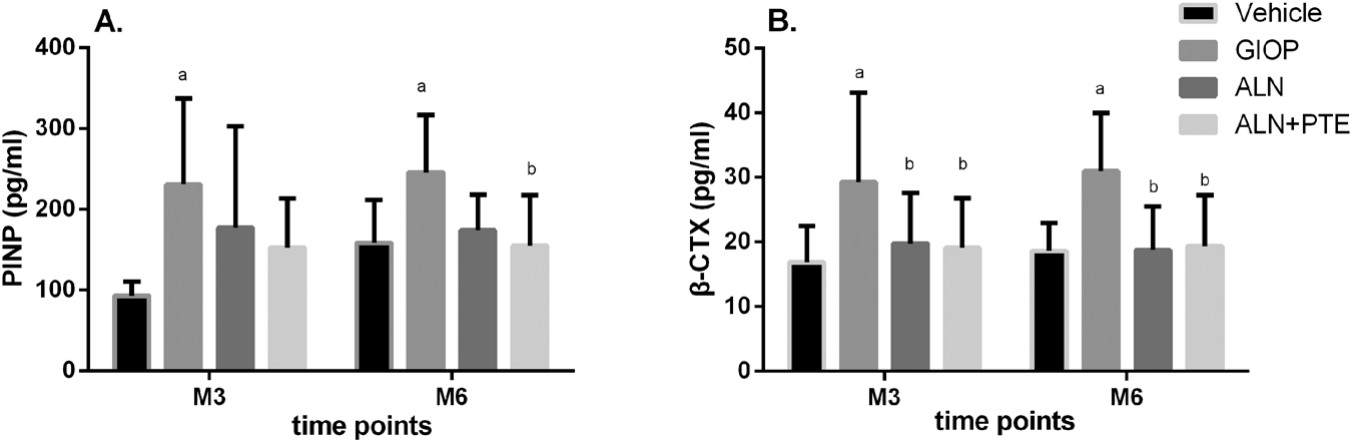
Changes in PINP and β-CTX levels. Values are the means ± SD. ^a^ *P* < 0.05 vs the vehicle group; ^b^ *P* < 0.05 vs the GIOP group; ^c^ *P* < 0.05 vs the ALN group.

Figure 3 shows the changes in serum marker levels over time. After DXM intervention (at M3), the GIOP group (compared to the Vehicle group) exhibited significantly higher levels of PINP and β-CTX. Compared with the GIOP group, the ALN+PTE group exhibited lower β-CTX and PINP levels, although the difference in PINP levels was no significant. Compared with the ALN group, the ALN+PTE group exhibited a lower PINP level, although the difference was not significant.

After DXM withdrawal (at M6), the GIOP group (compared to the vehicle group) exhibited significantly higher levels of PINP and β-CTX. Compared with the GIOP group, the ALN+PTE group exhibited lower β-CTX level and PINP level. Compared with the ALN group, the ALN+PTE group exhibited a lower PINP level, although the difference was not significant (Fig. 3).

### ALN+PTE mitigates histological damage during both the DXM intervention and withdrawal periods

**Figure 4:**
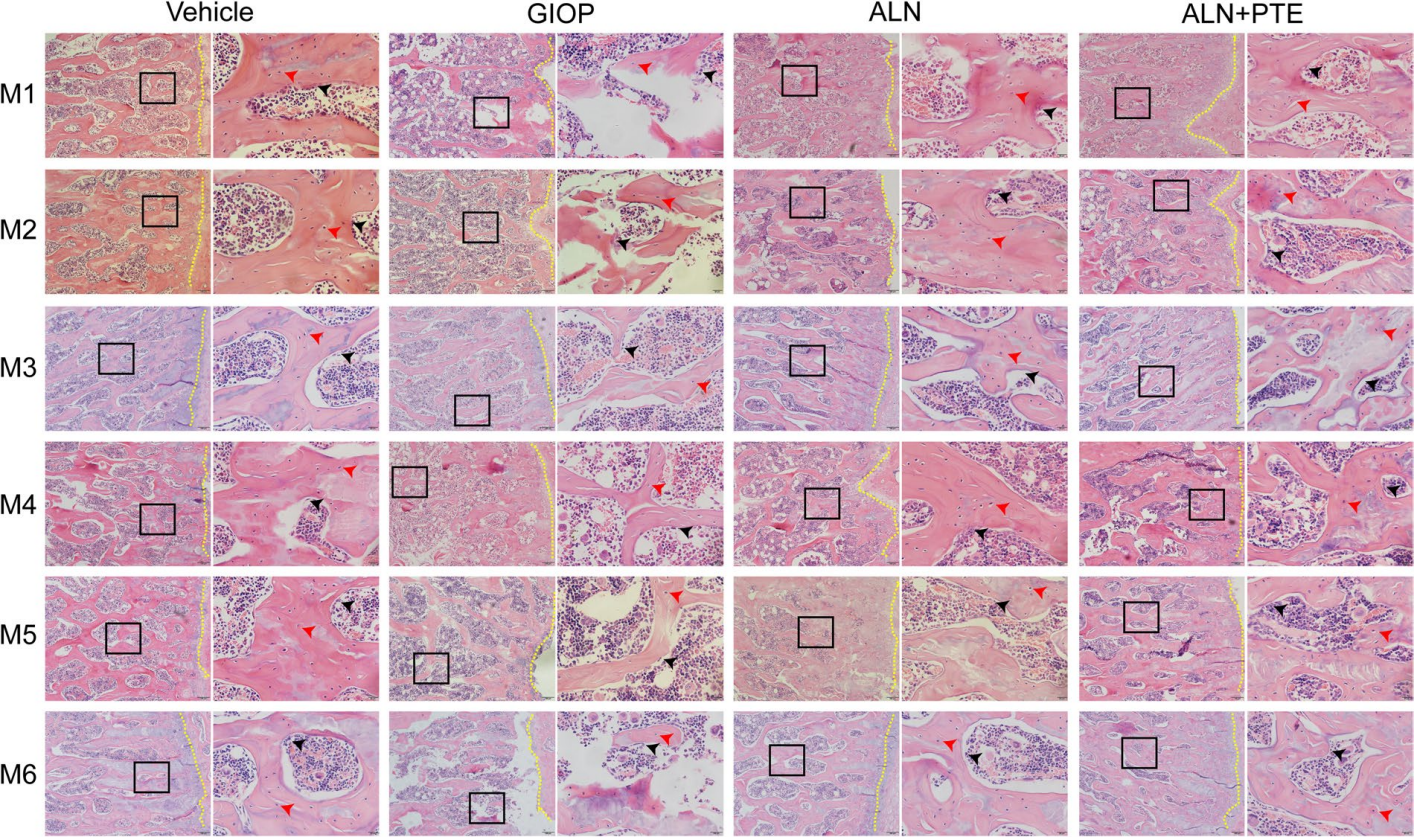
Changes of morphology in the L4 bone trabecular of rats in each group (H&E staining).The images under high power field of microscope (40×) were placed at on the left side of the images under low power field of microscope (4×). The yellow dotted line represents epiphyseal line; The red arrows represents osteocytes; The black arrows represents osteoblasts.

Figure 4 shows the morphological changes in the L4 trabeculae of the various groups. During both DXM intervention and withdrawal, the GIOP group (compared to the vehicle group) exhibited significantly increased trabecular spacing, poor trabecular continuity; significant trabecular absorption, perforation, and fracture; and reduced numbers of both osteocytes and osteoblasts. Compared with the GIOP and ALN groups, the ALN+PTE group exhibited notable improvements in terms of all of trabecular spacing, continuity, integrity, thickness, absorption, perforation, and fracture; and osteocyte/osteoblast numbers.

### ALN+PTE significantly increases osteoclast numbers during both the DXM intervention and withdrawal periods

**Figure 5:**
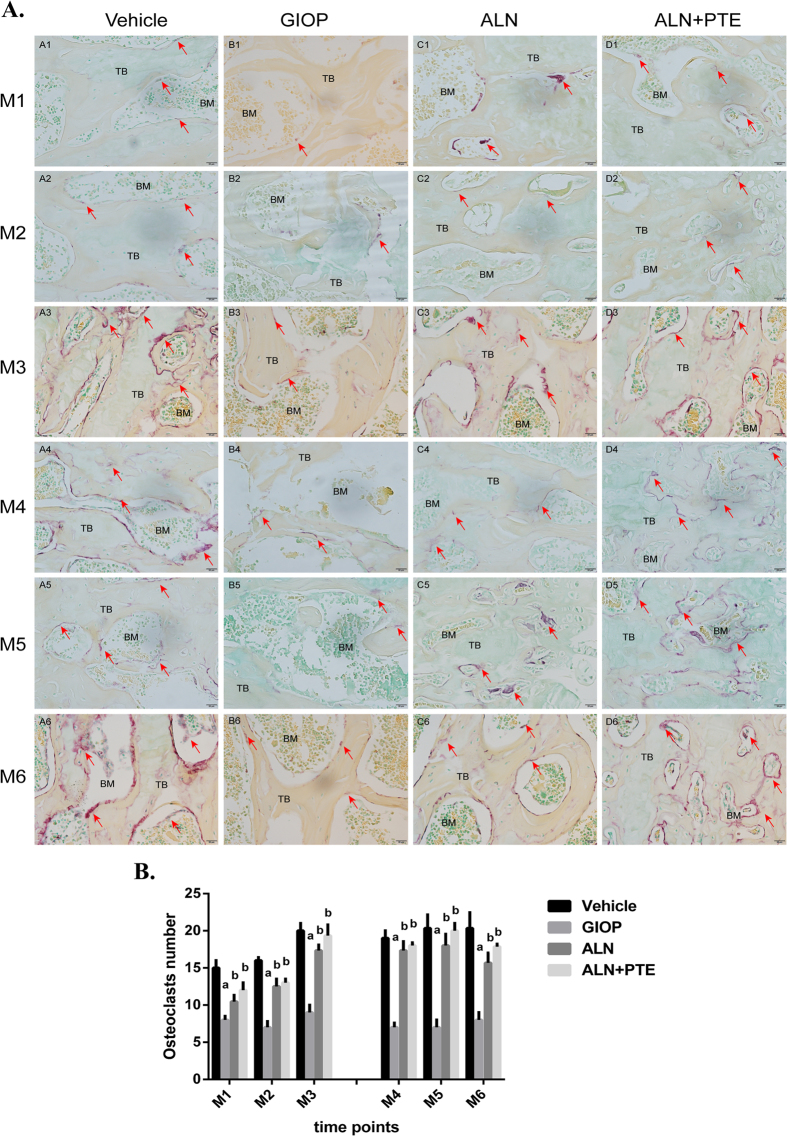
(**A**) Results of TRAP staining. 40× magnified images of L4 after TRAP staining, the multinuclear osteoclasts are stained red (red arrows) and the number of osteoclasts was quantified on four sections per specimen. “TB” represents trabecular bone; “BM” represents bone marrow. (**B**) Analysis of the numbers of osteoclasts. Values are the means ± SD. ^a^ *P* < 0.05 vs the vehicle group; ^b^ *P* < 0.05 vs the GIOP group; ^c^ *P* < 0.05 vs the ALN group.

Figure 5 show the numbers of tartrate-resistant alkaline phosphatase (TRAP)-positive osteoclasts in the L4 regions of the various groups, which also reflect the numbers of osteoblasts, because bone homeostasis is mediated by osteoblastic bone formation and osteoclastic bone resorption. During both the DXM intervention and withdrawal periods, the numbers of osteoclasts on the surfaces of the trabeculae were significantly lower in the GIOP group compared with the vehicle group at all time-points. Compared with the GIOP group, the ALN+PTE group exhibited significant increases in osteoclast numbers. Compared with the ALN group, the ALN+PTE group exhibited larger numbers of osteoclasts, although the difference was not significant.

### ALN+PTE promotes Runx2 mRNA expression to some extent during both the DXM intervention and withdrawal periods, and reduces CTSK mRNA expression during DXM withdrawal period

**Figure 6:**
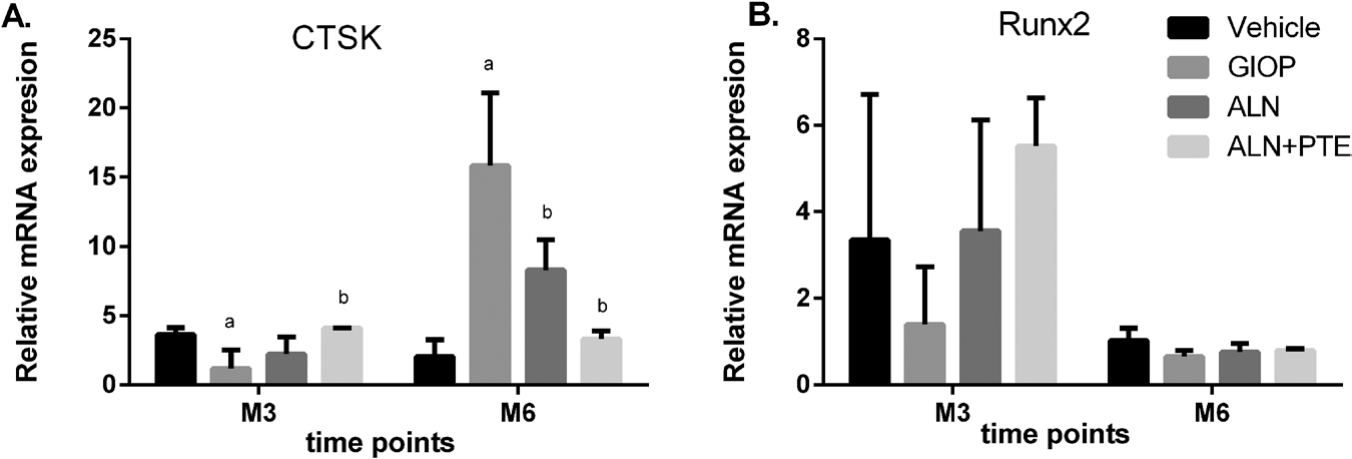
The mRNA expression levels of CTSK and Runx2. Values are the means ± SD. ^a^ *P* < 0.05 vs the vehicle group; ^b^ *P* < 0.05 vs the GIOP group; ^c^ *P* < 0.05 vs the ALN group.

The levels of mRNAs encoding CTSK and Runx2 were evaluated to explore how the ALN+PTE combination might function. After DXM intervention (at M3), CTSK mRNA expression was significantly down-regulated in the GIOP group. The Runx2 mRNA level was also down-regulated to some extent, but did not differ significantly from that of the vehicle group. After ALN+PTE treatment, the CTSK mRNA level was significantly up-regulated compared with the levels in the GIOP and ALN groups. The Runx2 mRNA level was also up-regulated to some extent, although the difference was not significant (Fig. 6).

After DXM withdrawal (at M6), the CTSK mRNA level in the GIOP group was significantly up-regulated. The Runx2 mRNA level was down-regulated to some extent, but did not differ significantly from that of the vehicle group. After ALN+PTE treatment, the CTSK mRNA level was significantly down-regulated compared with the GIOP and ALN groups. The Runx2 mRNA level was up-regulated to some extent, although the difference was not significant (Fig. 6).

### ALN+PTE group exhibits weaker CTSK immunostaining and stronger Runx2 immunostaining during both the DXM intervention and withdrawal periods

**Figure 7:**
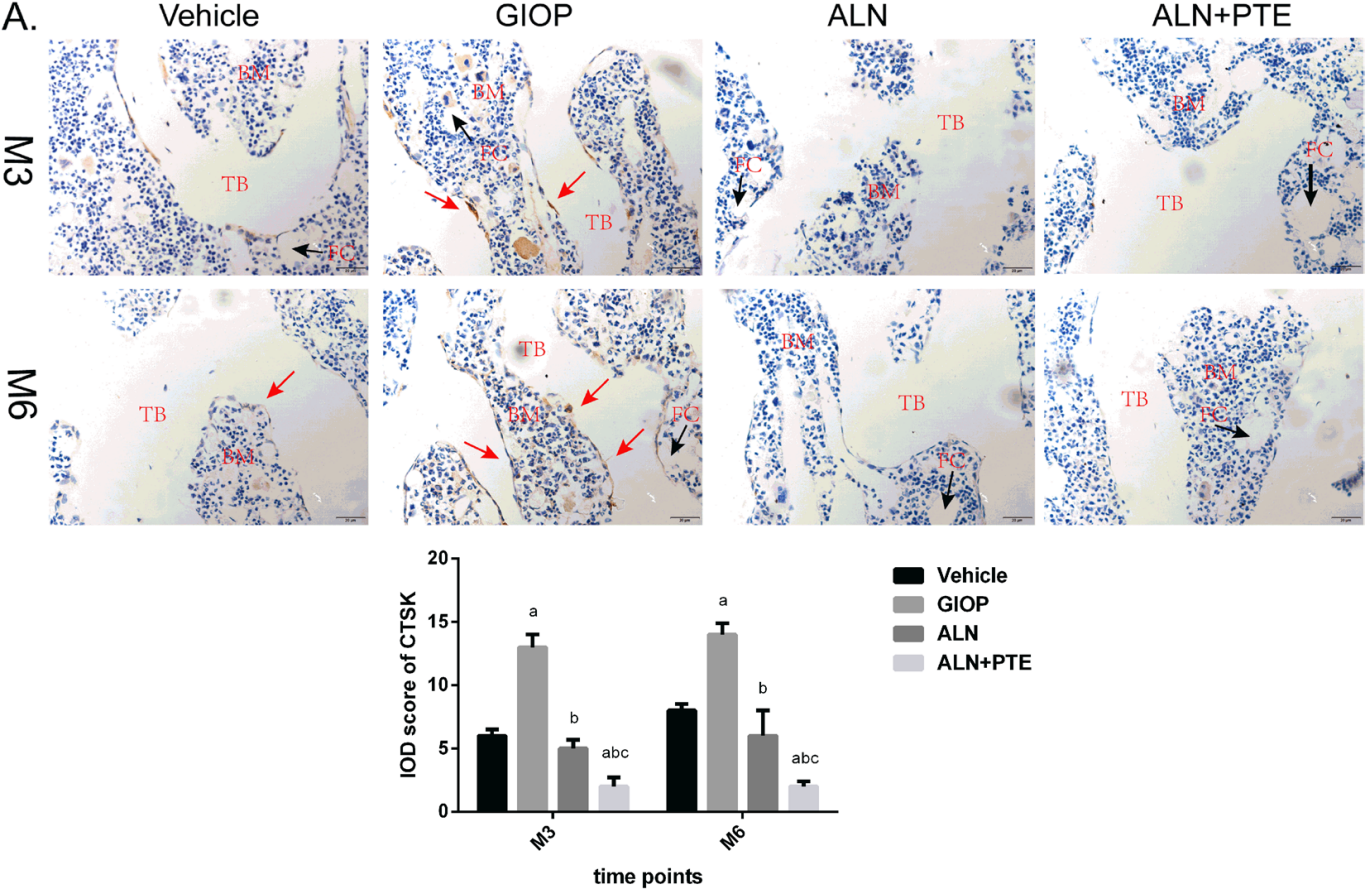
IHC was performed for evaluating protein expression level of CTSK (A). “TB” represents trabecular bone; “BM” represents bone marrow; “FC” represents fat cell. Values are the means ± SD. ^a^ *P* < 0.05 vs the vehicle group; ^b^ *P* < 0.05 vs the GIOP group; ^c^ *P* < 0.05 vs the ALN group.

**Figure 8:**
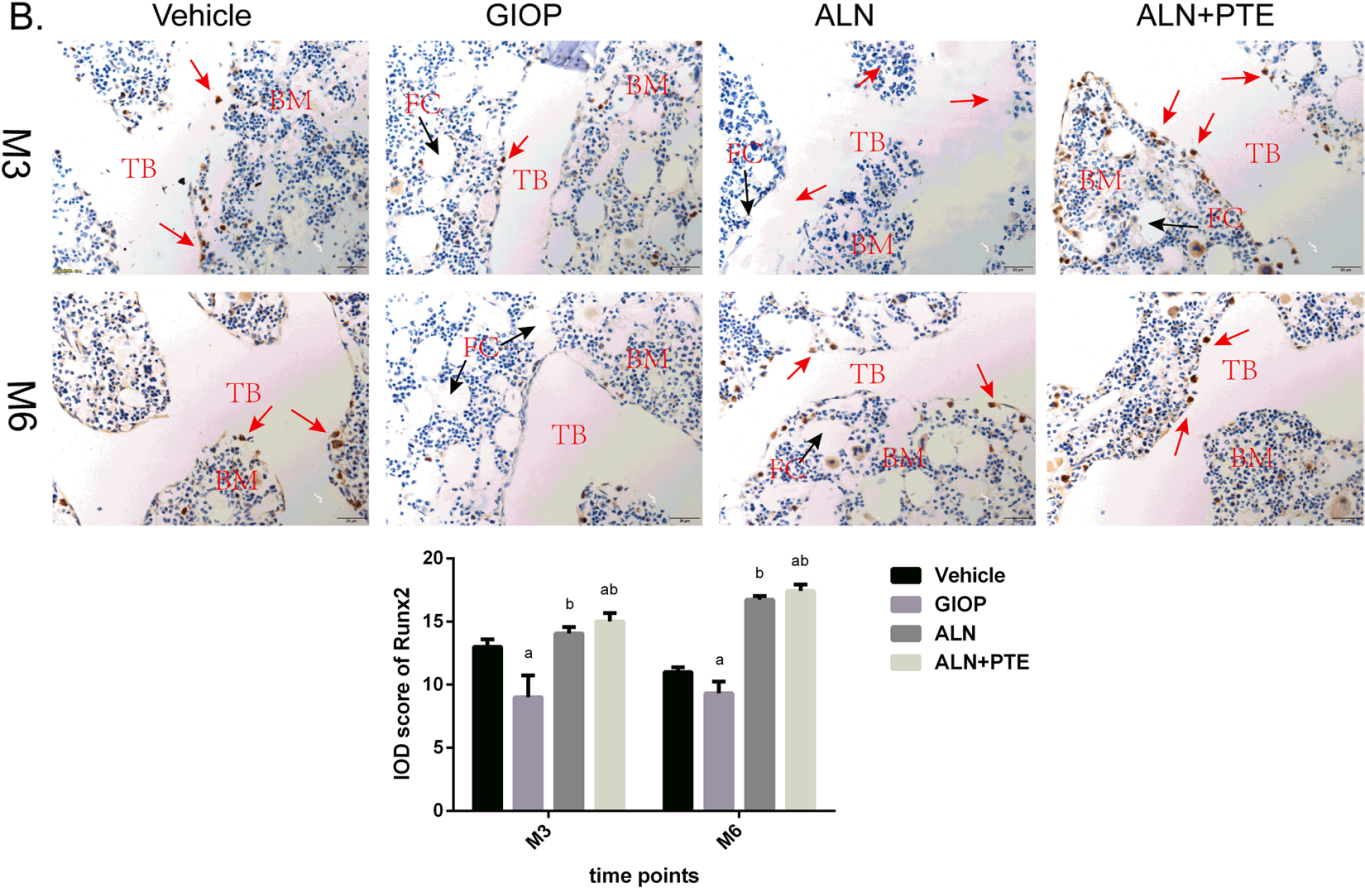
IHC was performed for evaluating protein expression level of Runx2 (B). “TB” represents trabecular bone; “BM” represents bone marrow; “FC” represents fat cell. Values are the means ± SD. ^a^ *P* < 0.05 vs the vehicle group; ^b^ *P* < 0.05 vs the GIOP group; ^c^ *P* < 0.05 vs the ALN group.

Immunohistochemical (IHC) assays were performed to determine the levels of CTSK and Runx2 proteins in the L4 vertebrae. After DXM intervention (time M3) and withdrawal (time M6), the GIOP group (compared to the vehicle group) exhibited stronger CTSK immunostaining, and weaker Runx2 immunostaining, at the trabecular surface. Compared with the GIOP and ALN groups, the ALN+PTE group exhibited weaker CTSK immunostaining and stronger Runx2 immunostaining (Figs 7 and 8).

After both DXM intervention (M3) and withdrawal (M6), the CTSK level was significantly higher and the Runx2 level significantly lower in the GIOP group compared with the vehicle group. The CTSK level was significant lower in the ALN+PTE group than the GIOP and ALN groups, and the Runx2 level was significantly higher than that of the GIOP group (Figs 7 and 8).

### ALN+PTE reduces CTSK protein expression and promotes Runx2 protein expression during both the DXM intervention and withdrawal periods

**Figure 9:**
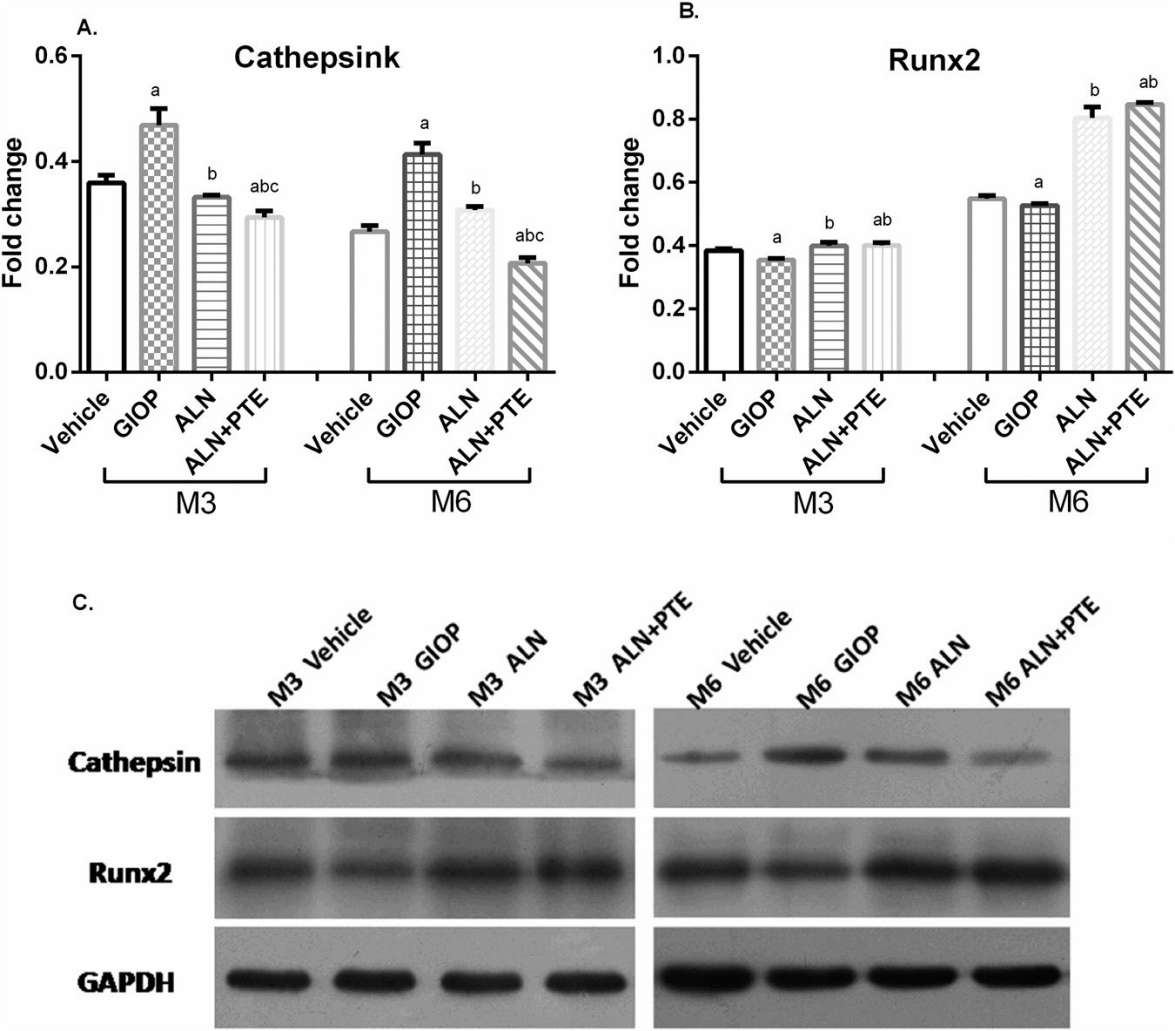
Protein expression levels of CTSK and Runx2 were analyzed by WB. Values are the means ± SD. ^a^ *P* < 0.05 vs the vehicle group; ^b^ *P* < 0.05 vs the GIOP group; ^c^ *P* < 0.05 vs the ALN group. The gels/blots displayed here are cropped, and without high-contrast (overexposure). The full-length gels and blots are included in a Supplementary Information file.

Both CTSK and Runx2 protein levels were analyzed by Western blotting. After both DXM intervention (M3) and withdrawal (M6), the GIOP group (compared to the vehicle group) exhibited significantly higher CTSK expression and significantly lower Runx2 expression. Compared with the GIOP and ALN groups, the ALN+PTE group exhibited significant lower CTSK expression. Compared with the GIOP group, the ALN+PTE group exhibited significantly higher Runx2 expression (Fig. 9).

## Discussion

We explored the synergistic actions of ALN and PTE *in vivo*, and present a new means treating GIOP. To our knowledge, this study is the first to show that ALN and PTE synergistically reduce GIOP *in vivo*. Also, for the first time, we have shown that the ALN+PTE combination reverses bone impairment in rats 3 months after GC withdrawal. Therefore, the combination of PTE and ALN may be a promising new anti-GIOP treatment.

BMD measurement is the gold standard in terms of GIOP evaluation and diagnosis, and contributes significantly to our understanding of GIOP pathogenesis as well as prevention, treatment, monitoring, and prognosis^14^. However, BMD measurement alone does not yield all required data; the pathological changes of GIOP develop mainly in cancellous bone. Such bone is composed of interconnected rods and plates forming a three-dimensional branched lattice^15^; BMD measurements do not reflect the stability or mechanical strength of cancellous bone^16–18^. Typically, osteoporosis reduces the extent of trabecular bone, and thins the bone; the intertrabecular spaces enlarged and the typical interconnected structure of trabecular bone is disrupted^19^. Trabecular perforations develop in the plate-like structures; the normal plate-like trabeculae may change to thinner rod-like structures, which may then disappear completely, increasing the risk of fracture^20^. Thus, assessment of microstructural changes in cancellous bone is essential when studying osteoporotic fractures and when evaluating anti-osteoporotic agents^19^. However, bone microstructure is often not evaluated in clinical practice. Bone mass and quality are useful measures of bone biomechanical properties^21–24^. These two indicators are often used to evaluate osteoporosis in animal models. In addition, bone histomorphology reflects the distribution and numbers of trabeculae, but not the numbers of osteoblasts, osteoclasts, or osteocyte; or marrow cavity status. The latter parameters are useful indicators of bone impairment, increasing our understanding of the underlying pathogenesis and providing the experimental basis for clinical treatment.

We found that DXM induced serious bone mass loss, comprised bone microarchitecture, reduced bone strength, and impaired the histological profile. These results were consistent with those of previous studies^25, 26^, thus validating the GIOP model. Also, GC withdrawal for 3 months did not improve bone impairment in rats with GIOP, as also found in previous studies^13^. During the GC intervention period, ALN+PTE increased the BMD and BMC, improved bone microarchitecture, increased bone strength, and reversed histologically evident damage. During the GC withdrawal period, ALN+PTE exerted similar effects. ALN+PTE was better than ALN alone in terms of improvements evident upon micro-CT examination, biomechanical testing, and histomorphological examination. Our findings establish the experimental basis for the clinical integration of traditional Chinese and Western medicine.

Presently, PINP is the most sensitive markers of bone formation^27^ and β-CTX one of the most reliable markers of bone resorption^28^. Both markers are used to evaluate homeostasis within bone microenvironment. Consistent with previous studies^13, 25, 26^, we found that both DXM intervention and withdrawal induced high-level bone turnover, increasing both bone formation and resorption. The monitoring of changes in PINP and β-CTX levels induced by bone-active drugs plays an important role in characterizing drug effects on the basic bone multicellular units, and bone remodelling^29^. We found that during both GC intervention and withdrawal, ALN+PTE reduced the levels of bone metabolic markers PINP and β-CTX, showing that ALN+PTE played a key role in reversing imbalances in bone formation and resorption. ALN+PTE improved all of GC-induced bone loss, microstructural impairment, biomechanical properties, and histomorphological pathology.

All of skeletal development, bone matrix mineralization, and bone homeostasis are closely regulated at the gene and protein levels. We thus measured the mRNA and protein levels of determinants of bone formation and resorption, to define the therapeutic target(s) of ALN+PTE. Runx2 is a major osteoblast transcription factor, promoting bone formation by interfacing with the canonical Wnt signaling pathway^30, 31^. CTSK is abundant in osteoclasts, promoting bone matrix degradation^32–34^. Therefore, we evaluated Runx2 and CTSK expression levels and confirmed that 3 months of GC treatment promoted bone resorption and inhibited bone formation; GC withdrawal for a further 3 months did not reverse such imbalances in bone homeostasis. ALN+PTE significantly reduced CTSK expression and promoted Runx2 expression after both DXM intervention and withdrawal, and inhibited bone resorption better than did ALN treatment alone, suggesting that ALN and PTE exerted significant synergistic effect when countering the bone impairment evident after GC withdrawal. Both CTSK and Runx2 may be the key targets of ALN and PTE combination. However, the CTSK mRNA levels were inconsistent with those of the CTSK protein after 3 months of GC intervention. This may be attributable to microRNA-based regulation; further work is needed.

We thus first showed that ALN and PTE synergistically reduced the effects of GIOP in the rat spine, and then identified the potential therapeutic targets. However, our study had several limitations. First, although Runx2 and CTSK were found to be the effective therapeutic targets, other genes and protein may also be involved and should be identified in future work. Second, although some differential expression of genes and proteins was evident, we did not use gene knockout or overexpression to further elucidate potential mechanisms of drug action. Our findings require clinical and experimental verification.

## Methods

### Experimental animals and study design

Female Sprague Dawley rats (n = 128) 3 month of age (from Guangzhou University of Chinese Medicine) were housed in cages under standard laboratory conditions (22–25 °C and an atmospheric pressure of 25 kPa) in the animal room of the First Affiliated Hospital of Guangzhou University of Chinese Medicine (approval no. SYXK [Yue] 2013–0092) under a 12-h/12-h light/dark cycle (lights on at 08:00) with free access to a standard rodent diet and water.

**Figure 10:**
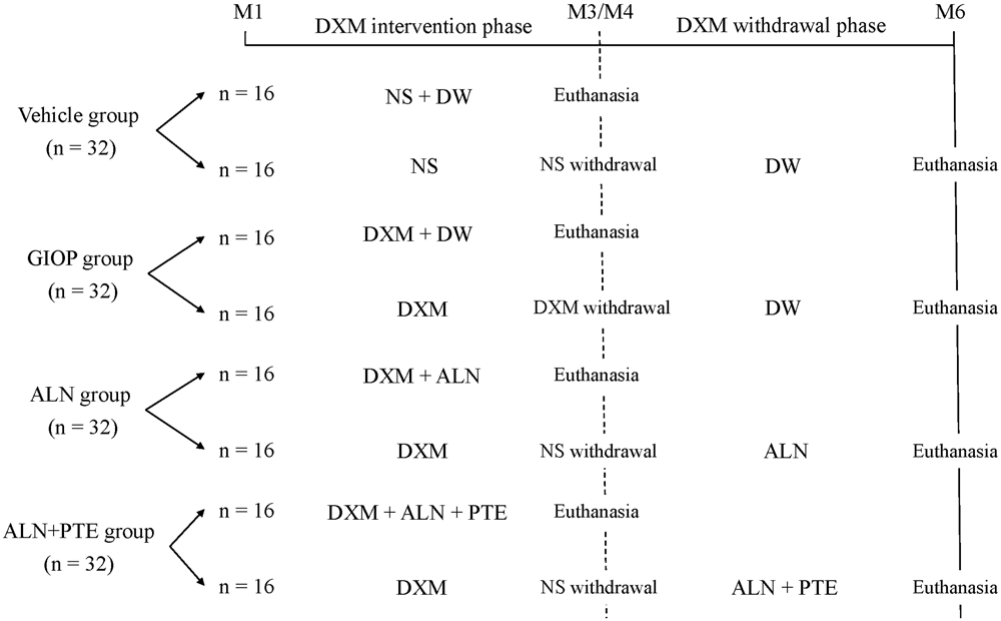
Animal grouping and intervention approach in this study. “NS” represents normal saline (received the injection at a dose of 0.6 mg/kg body weight, twice per week), “DW” represents distilled water (received oral at a dose of 5 ml/kg, once a day); “DXM” represents dexamethasone-21-isonicotinate (received the injection at a dose of 0.6 mg/kg body weight, twice per week); ALN represents alendronate (received oral at a dose of 1 mg/kg, once a week); PTE represents extracts from plastrum testudinis (received oral at a dose of 30 mg/kg, once a day).

After undergoing 7-day of adaptation, the rats were randomly assigned to one of four groups: A vehicle group, a GIOP group, an ALN group, and an ALN+PTE group (n = 32/group). Each group was further divided into two experimental phases (n = 16/phase), including DXM intervention phase and DXM withdrawal phase. During the DXM intervention period (M1-M3), the vehicle group animals were injected daily with the vehicle, whereas the other groups received subcutaneous injection of 0.6 mg/kg DXM every 3 days for 3 months. And during this period, 16 rats from each of the vehicle and GIOP groups received distilled water (DW) orally every day, whereas 16 rats from each of the ALN and ALN+PTE groups received oral ALN at 2 mg/kg three times weekly or ALN and PTE combination at 30 mg/kg (each) daily. During the DXM withdrawal period (M4-M6), the remaining 16 rats from each group were gavaged decribed above for the entire 3 months (Fig. 10).

Bone samples (lumbar vertebrae) devoid of soft tissues were isolated after euthanizing the rats. En bloc lumbar vertebrae 1–3 (L1–3) samples were stored at −20 °C prior to dual-energy X-ray absorptiometry (DXA). Lumbar vertebrae 2 (L2) samples obtained from L1–3 after DXA were fixed in 4% (w/v) phosphate-buffered paraformaldehyde prior to micro-CT and biomechanical analysis. Lumbar vertebrae 4 (L4) were transferred to 4% (w/v) phosphate-buffered paraformaldehyde prior to TRAP staining and IHC. Blood samples were collected and stored at −20 °C prior to assessment of bone turnover markers, including serum PINP and β-CTX levels. Lumbar vertebrae 5 (L5) and 6 (L6) samples were preserved at −80 °C prior to evaluation of mRNA and protein expression levels.

### Ethical approval

All experimental procedures were approved by the Ethic Committee of the First Affiliated Hospital of Guangzhou University of Chinese Medicine (approval no. 20130425). All care procedures followed the Guideline for the Care and Use of Laboratory Animals published by the United States National Institutes of Health.

### Bone mass measurement by DXA

The BMD (g/cm^2^), BMC (g), and AREA (cm^2^) of the en-bloc L1-3 samples were measured by DXA using a small-animal high-resolution collimator (Discovery A/SL/W/C; Hologic, Bedford, MA, USA). Regions of interest were marked over the entire L1-3 area All analysis employed the small animal evaluation software supplied with the collimator (version 13.2:3; Hologic); the device was calibrated at the beginning of the experiment.

### Bone microarchitecture assessed by micro-CT

The structural parameters of L2 samples were quantified with the aid of a cone beam-type desktop micro-CT unit (μCT80; Scanco Medical, Brüttisellen, Zurich, Switzerland) running inbuilt software (μCT80 Evaluation Program v. 6.5-1; Scanco Medical). The analytical parameters were set to 55 kVp and 80 μA, and the spatial resolution was 14 mm in all directions. After scanning, cancellous vertebral bone was chosen as the volume of interest, restricted to internal vertebral region. The trabecular and cortical bones were identified by drawing cylinder contours (diameter, 2 mm) with the aid of CT software.

We measured BS/TV (mm^-1^), BV/TV (%), Tb.N (1/mm), Tb.Th (mm), Tb.Sp (mm), and vBMD (mg HA/cm^3^). Reconstructed three-dimensional images were obtained via multiplanar reformation.

### Bone biomechanics as evaluated by compression testing

Bone mechanical quality was assessed using a materials testing device (ElectroPuls E1000 test system; Instron Corp., Norwood, MA, USA) to evaluate L2 samples after micro-CT testing. Both endplates of the vertebral body, and the appendix, were removed to obtain central cylinders with planoparallel ends approximately 5 mm in height. All vertebrae were then tested along the longitudinal axes at a constant compression speed of 1 mm/min.

Load-displacement curves were plotted using inbuilt software (Bluehill 3; Instron Corp.) and yielded compressive strength (in N), compressive stiffness (in N/mm), compressive displacement (in mm), and energy absorption capacity (in J).

### ELISA of serum markers

Sera were thawed and analyzed via an enzyme-linked immunosorbent assay (ELISA) in terms of the levels of PINP and β-CTX (intra-assay precision: coefficient of variation [CV] < 8%; inter-assay precision: CV < 10%) according to the manufacturer’s protocols (Cusabio, Wuhan, Hubei, China).

### Bone morphology as observed by H&E and TRAP staining

After removal of surface soft tissue, L4 samples were decalcified in 10% (w/v) EDTA (disodium salt) for about 2 months, embedded in paraffin, and cut into 5-μm-thick serial sagittal slides. Some slides were stained with hematoxylin and eosin (H&E) to allow of descriptive analysis, and the rest with TRAP (Sigma, St. Louis, MO, USA) to identify osteoclasts on the bone surface. All slides were covered with neutral resin (Shanghai, China, batch no: 20141123) and observed under the microscope (BX53, Olympus Corp., Japan). Photographs were analyzed with the aid of CellSens Dimension software (version 510-UMA-CellSens19.0-krishna-ch-00-01; Germany).

### mRNA expression detected by RT-PCR

The levels of expression of mRNAs encoding CTSK and Runx2 were evaluated by RT-PCR. Total RNAs from L5 vertebrae were extracted using the protocol of the Takara MiniBEST Universal RNA Extraction Kit (catalogue no. 9767, Takara, Japan). Sixty microliter amounts of reaction solution (10 μL of solution can contain up to 500 ng of RNA) were subjected to reverse transcription using the PrimeScriptTM RT Master Mix (Perfect Real Time) kit (catalogue no. RR036A, Takara). Real-time analysis was performed with the aid of a Bio-Rad iQ5 PCR cycler using a SYBRR Premix Ex TaqTMII (TilRNaseH Plus) kit (catalogue no. RR820A, Takara). β-actin served as the internal standard. 2 ul cDNA was amplified with the special primer as follow: 5′-ACTCTGAAGACGCTTACCCG-3′ and 5′-CCTTTGCCGTGGCGTTATAC-3′ for CTSK; 5′-CAGACCAGCAGCACTCCATA-3′ and 5′-GCTTCCATCAGCGTCAACAC-3′ for Runx2; 5′-GGAGATTACTGCCCTGGCTCCTA-3′ and 5′-GACTCATCGTACTCCTGCTTGCTG-3′ for β-actin. Relative gene expression levels were quantified using the 2^−DΔCt^ method.

### Protein expression levels as revealed by IHC

We used a routine method to rehydrate the slides prepared as described above and blocked non-specific binding by addition of hydrogen peroxide. The slides were then incubated with anti-Cathepsin K antibody (catalogue no. ab19027; Abcam, Cambridge, MA, USA) and anti-RUNX2 antibody (ab76956, Abcam) at 4 °C overnight followed by incubation with HRP-conjugated goat anti-rabbit IgG antibody (DAKO Inc., Denmark) at room temperature for 30 min. The slides were counterstained with haematoxylin at room temperature for 6 min, covered with neutral resin, and observed under a microscope (BX53, Olympus). The images were photographed and analyzed using CellSens Dimension software (version 510-UMA-CellSens19.0-krishnach-00-01). The integrated optical densities (IOD) were semiquantitatively analyzed with the aid of Imaging Pro Plus 6.0 software (Media Cybernetics, Rockville, MD, USA).

### Western blotting of Proteins

L6 vertebrae frozen in liquid nitrogen were pulverised in a mortar followed by homogenization in ice-cold radio immunoprecipitation assay (RIPA) buffer (Beyotime, Nantong, Jiangsu, China). Total protein concentrations were determined using a BCA Protein Assay kit (Beyotime) according to the manufacturer’s instructions. Samples containing approximately 80 μg of protein were subjected to sodium dodecyl sulphate polyacrylamide gel electrophoresis (SDS-PAGE), and the proteins transferred to polyvinylidene fluoride (PVDF) membrane (EMD Millipore, Billerica, MA, USA). After blocking non-specific binding with a 5% (w/v) non-fat dry milk solution for 1.5 h at room temperature, the membranes were incubated overnight at 4 °C with the following primary antibodies: an anti-RIP1 monoclonal antibody (Abcam), an anti-RIP3 polyclonal antibody (Abcam), and an anti- β-actin monoclonal antibody (Santa Cruz Biotechnology, Santa Cruz, CA, USA). Protein bands were visualized using an ECL detection kit (Beyotime) and expression levels were densitometrically quantified using Image Lab version 2.1 (Bio-Rad). The values for each protein were normalized to those of β-actin.

### Statistical analysis

All statistical analyses were performed with the aid of SPSS version 19.0 software (SPSS Inc, Chicago, IL, USA). All data were checked for normality and homogeneity of variance and were expressed as means ± standard deviation (SD). Changes in all of BMD, BMC, AREA, bone microarchitecture, and biomechanics were subjected to two-way analysis of variance (ANOVA). One-way ANOVA was used to make all comparisons of serum markers and relative mRNA expression levels among groups. When ANOVA identified a significant difference, intergroup comparisons were performed using the least significant difference (LSD) test. In all analyses, *P* < 0.05 was considered to reflect statistically significance.

## Electronic supplementary material

Supplementary Information
