# Supplementary material for: Promotion effect of extracts from plastrum testudinis on alendronate against glucocorticoid-induced osteoporosis in rat spine
Source: Sci Rep. 2017 Sep 6;7:10617. doi: 10.1038/s41598-017-10614-5 (PMC5587701; doi:10.1038/s41598-017-10614-5)
Supplement: Supplementary file 1 — Supplementary Information [file 41598_2017_10614_MOESM1_ESM.pdf]

Promotion effect of extracts from plastrum testudinis on alendronate against glucocorticoid-induced osteoporosis in rat spine

Hui Ren[1], Gengyang Shen[1], Jingjing Tang[2], Ting Qiu[1], Zhida Zhang[1], Wenhua Zhao[1], Xiang Yu[1], Jinjing Huang[1], De Liang[2], Zhensong Yao[2], Zhidong Yang[2], Xiaobing Jiang[2,3,*]

1 Guangzhou University of Chinese Medicine, Guangzhou, China, 510405.

2 Department of Spinal Surgery, The First Affiliated Hospital of Guangzhou University of Chinese Medicine, Guangzhou, China, 510405.

3 Laboratory Affiliated to National Key Discipline of Orthopaedic and Traumatology of Chinese Medicine, Guangzhou University of Chinese Medicine, Guangzhou, China, 510405.

Supplementary Information

The full-length gels and blots are as follows:

A:

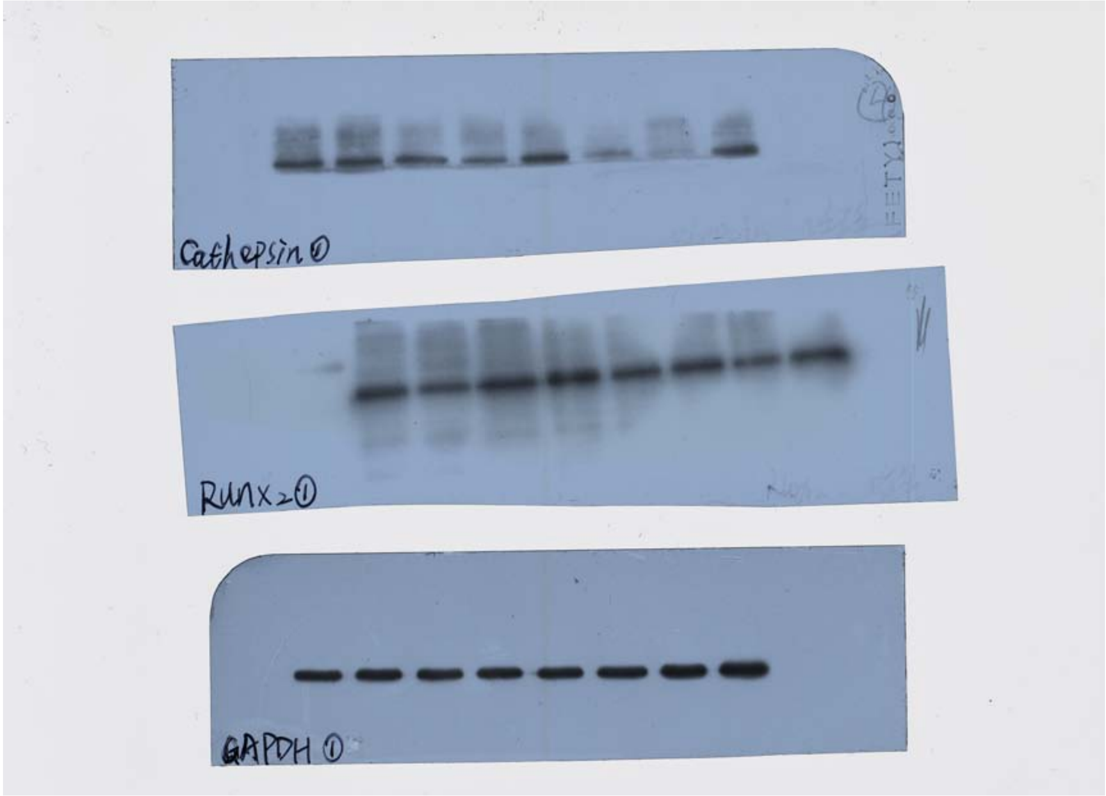

B:

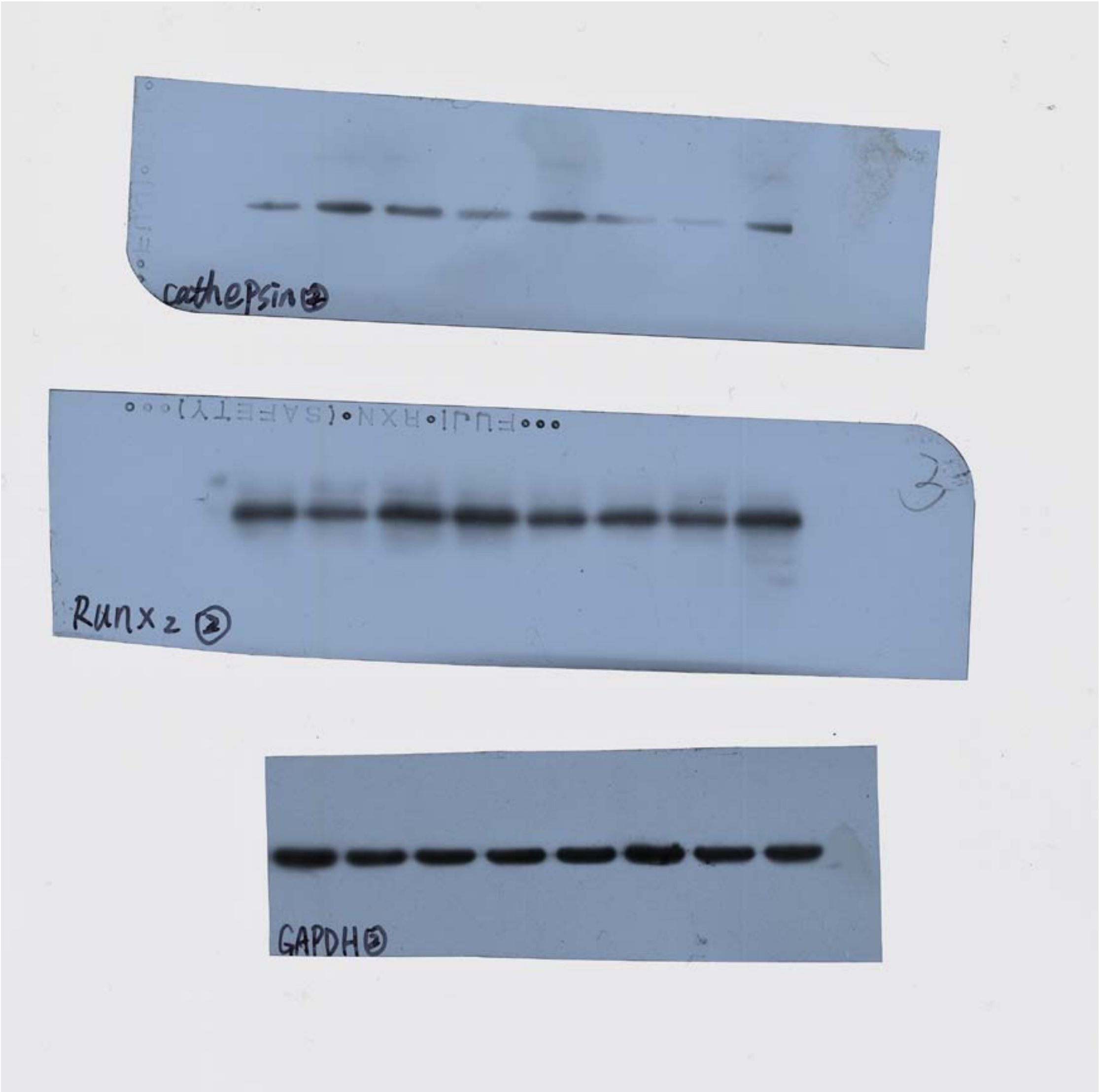

Note: These are the full-length gels and blots. “A” represents the CTSK and Runx2 protein levels after DXM intervention (M3), and “B” represents that after DXM withdrawal (M6). We cropped the previous four gels and blots, because the back four gels and blots belonged to other experiments. The gels and blots are without high-contrast (overexposure).
